# Challenges for the Implementation of the First Large-Scale Rheumatic Heart Disease Screening Program in Brazil: The PROVAR Study Experience

**DOI:** 10.5935/abc.20170047

**Published:** 2017-04

**Authors:** Julia Pereira Afonso dos Santos, Gabriel Assis Lopes do Carmo, Andrea Zawacki Beaton, Tainá Vitti Lourenço, Adriana Costa Diamantino, Maria do Carmo Pereira Nunes, Craig Sable, Bruno Ramos Nascimento

**Affiliations:** 1 Hospital das Clínicas da Universidade Federal de Minas Gerais, MG - Brazil; 2 Childrens National Health System, Washington - EUA

**Keywords:** Rheumatic Heart Disease, Mass Screening, Echocardiography, Child, Adolescent

## Introduction

Rheumatic Heart Disease (RHD) is the cardiac consequence of acute rheumatic fever (ARF), an inflammatory disease triggered by streptococcal pharyngitis. Although the prevalence of RHD has decreased in high-income countries, lack of social and economic development and poor primary prevention - mainly in in low- and middle-income countries - perpetuate an environment where RHD remains endemic. It is estimated that RHD continues to affect nearly 33 million people worldwide.^1^ According to the World Health Organization (WHO), RHD is responsible for 1-1.5% of all cardiovascular deaths and 3-4% of cardiovascular Disability-Adjusted Life Years (DALYs).^2^ In Brazil, according to the Unified Health System (SUS), there were 26,054 hospital admissions for ARF (45% with cardiac compromise) between 2008 and 2015, and the total cost to SUS was US$3.5 million, a number that is most likely underestimated.^3^

The main burden of RHD to public health systems consists of repeated hospital admissions and cardiac surgeries in the following decades after initial cardiac damage. If RHD is detected in its early stages, secondary prophylaxis (regular penicillin injections) can be initiated to prevent new episodes of ARF, avoiding further valve damage and progression of RHD. In high prevalence regions, RHD meets the traditional screening criteria defined by Wilson and Jungner,^4^ although the long-term clinical significance of latent RHD is not entirely clear. Previous studies have demonstrated, however, that in 38 to 68% of asymptomatic RHD patients, echocardiographic findings show that abnormalities persist, and progress in 4 to 16%,^5^ reinforcing the importance of early diagnosis in susceptible populations.

The PROVAR study (*Programa de Rastreamento da Valvopatia Reumática -* Rheumatic Heart Disease Screening Program) is the first large-scale echocardiographic screening program in Brazil, using echocardiography to estimate the prevalence of latent RHD in asymptomatic children between 5 and 18 years old attending public schools of underserved areas of the cities Belo Horizonte, Montes Claros and Bocaiúva, in the Brazilian State of Minas Gerais. Minas Gerais is the second most populous Brazilian state (>20 million inhabitants) and has a large territory, great geographical diversity and is marked by economic discrepancies between its different regions. This project is a clinical and research collaboration between the University Hospital of *Universidade Federal de Minas Gerais* (UFMG), Brazil, and the Children's National Health System (CNHS) in Washington DC, United States of America (USA).

### Implementation of the study

**Figure 1:**
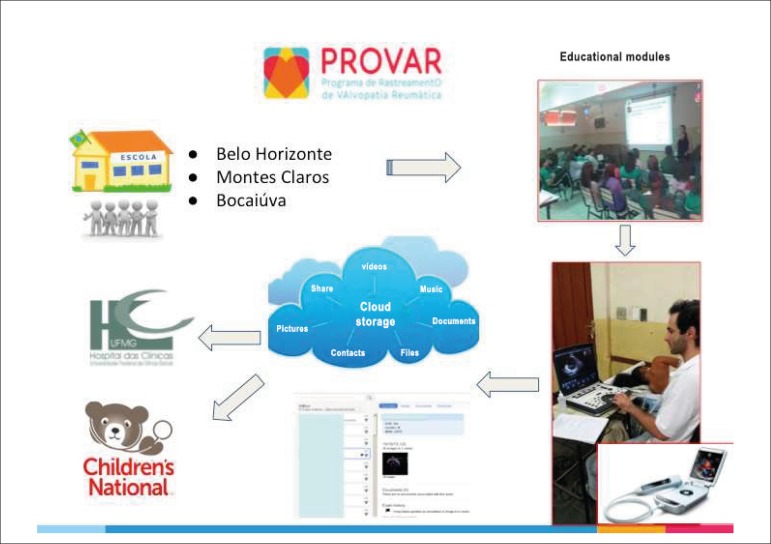
Operational flowchart of the PROVAR study: a) selection of schools in low-income areas; b) educational process; c) acquisition of echocardiographic images by non-physicians, using portable and handheld echo machines; d) image upload to cloud computing solutions with image viewing and measurement capability (LifeImage® (Newton, MA, USA) and ViTel Net®, (McLean, VA, USA)) for DICOM images from standard portable machines and secure Dropbox® plus dedicated Gateway® software for VSCAN; e) download and interpretation via telemedicine by cardiologists in Brazil and the United States (Sable, C. and Nascimento, B.R.).

The regulatory process started in the end of 2013, and the study was approved by the institutional review boards of UFMG and CNHS, as well as the State Boards of Health and Education. Legal sectors then analyzed the proposal and approved it, and there was no extra cost for the government or patients. The Department of Education selected schools with the highest socioeconomic vulnerability, with a special interest in areas with limited access to healthcare. Prior to screening, an educational curriculum was implemented, including lectures and printed material, for students and their parents, teachers and school staff regarding the importance of streptococcal pharyngitis, ARF and RHD. Parents were asked to sign an informed consent form - a requirement of Brazilian research regulations. Non-physician personnel started echocardiographic screening in July 2014 after a 12-week hands-on training supervised by an expert cardiologist and online RHD education modules (WiRED International, http://www.wiredhealthresources.net/EchoProject/index.html). They used portable (GE Vivid Q^®^) and handheld (GE VSCAN®) machines. Images were uploaded into dedicated cloud computing systems or Dropbox® and remotely interpreted by experienced cardiologists at UFMG (board certified by the Brazilian Society of Echocardiography) and CNHS using telemedicine resources (Figure 1).^6^ We used the World Heart Federation criteria for the diagnosis of RHD.^7^ Two experts blindly interpreted 10% of all acquired studies including 100% of the studies initially flagged as abnormal. In case of discrepancies during this process, a third expert blindly reviewed the images and a consensus diagnosis was reached.

When abnormalities were detected during the screening echocardiogram, a medical student called the child's parents to schedule a follow-up standard exam, which was performed by an experienced pediatric cardiologist at the university's hospital. Borderline RHD cases had a clinical consult and a follow up echocardiogram scheduled within one year. For patients found to have definite RHD based on the screening and follow-up echocardiogram, in addition to the referral to a specialized outpatient clinic, penicillin prophylaxis was initiated and more frequent follow-up echocardiograms were recommended based on the observations of subsequent visits.

### Initial results

**Figure 2:**
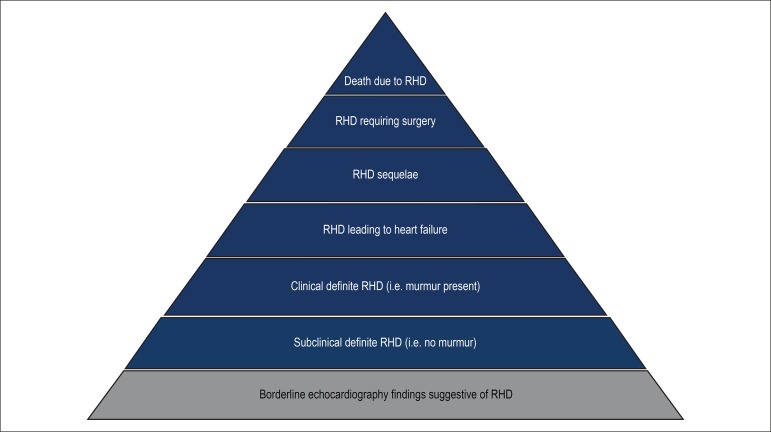
Progressive presentation of Rheumatic Heart Disease over time (adapted by Carmo, G.A.L. from Zühlke LJ and Steer AC.^5^).

The PROVAR team has educated over 20,000 children on RHD, and has performed over 9,000 echocardiograms in 32 schools.^8^ In the published analysis of 5,996 asymptomatic children from 21 schools, the overall prevalence of RHD was 4.2%, including 3.7% borderline (N = 221) and 0.5% definitive (N = 30). The inclusion of borderline patients as positive screening may raise some doubts, considering the available data. However, we believe this should be done, since this group seems to have increased the risk of progression to clinical RHD (Figure 2).^5,9^ Children with cardiovascular symptoms (self-reported or reported by teachers/parents) that eventually presented to the program were directly referred to tertiary care. It is important to note that non-physician personnel performed the screening echocardiograms. Only physicians are allowed to perform and read echocardiograms in Brazil, and we were able to conduct the study because it was a research protocol.

The accuracy of these non-physicians (nurses, technicians and medical students) for basic interpretation of simplified screening exams was tested and had good results: overall sensitivity of 83% and specificity of 85%.^10^ Moreover, the educational process has proven to be effective, with a median 20% increase in knowledge about RHD, evaluated through structured pre- and post-tests applied to more than 1,100 school children.^11^

### Main challenges

There were several challenges during the implementation and conduction of the PROVAR study. During the initial ethics approval - which took almost 90 days in Brazil - some resistance from the Board of Education and Health was observed, mainly related to research procedures and the impact on the school routine. Some modifications to the consent forms were required, which took an extra 4 months. There was also questioning by the Board of Health, related to concerns about task-shifting (which could be questioned by medical and nursing councils) and the impact of screening on the population: referral strategies, availability of penicillin for all positive cases - considering the shortage observed in Brazil - and information for families with children with positive exams to avoid stigmatization. 

During the initial steps of the field study, the PROVAR team faced several challenges. At first, the main challenges were related to the lack of involvement of school representatives with the program and lack of understanding possible benefits. Overall, there was low parental engagement with the project with the poorest attendance to educational sessions seen in the lowest socioeconomic status areas. The proportion of signed informed consents was low (about 35%: 5,996 out of 17,000 children)^8^ especially among older students, even after the educational process. Also, some school principals refused to participate despite approval by the ethics committee and government authorities. Finally, organizing the kids for screening, especially the youngest age groups, was a challenge: removing the children from the classroom, getting them to undress/dress and organized in a line, collecting demographic data, etc. Follow-up meetings with the Boards of Education and local delegates were scheduled on a regular basis to assess weaknesses and special needs of different communities, and to encourage school staff to support the project locally. Screening in primary care centers with population education through the Family Health Program - a plan for the following years - may also be an effective strategy to increase the scope of the program.

There were also several challenges for follow-up visits. Phone contact with parents was often not possible because the children did not have the contact information, numbers changed frequently, and schools are not always authorized to disclose contact information. In addition, 35% of families failed to show up for scheduled appointments. This may presumably be, in part, due to patients being asymptomatic and families not being convinced of the importance of monitoring. Based on conversations with parents who have not returned for the follow-up, our team hypothesizes that financial constraints, living in distant neighborhoods and impossibility to miss a workday also contributed to the lack of compliance with recommendations for follow up in the study protocol. However, data about the reasons for the low compliance were not systematically collected. Some strategies, such as subsequent educational calls and flexible follow-up dates have been recently tested, with relative success. There is also a plan to provide follow-up echocardiograms in the schools, immediately after the screening process, to improve follow-up and referral rates. Additionally, educational and public awareness materials for different scenarios are under development, considering the questions posed by the families.

### Future directions

Fighting RHD is a challenge for Brazil. Understanding the burden of the disease and how it affects the health system is the primary objective of PROVAR. The PROVAR program will continue to screen for RHD in underserved areas, but we are now expanding our efforts to private schools, where students' socioeconomic conditions are much better. A much lower prevalence is anticipated in this "control" population.

Authorities should be prepared to effectively eradicate RHD. To do so, we believe ARF and RHD should be included in the list of notifiable diseases, since these conditions have a considerably high incidence in Brazil. Group A streptococcal infections (triggers for ARF) are communicable from patient to patient and transmission can be eliminated with eradication.^12^ Also, by identifying early RHD, secondary prophylaxis can be initiated to prevent disease progression and late consequences such as heart failure, endocarditis, arrhythmias, stroke and heart valve surgeries.

The next steps of PROVAR are related to the diagonal integration of RHD screening in primary care. This strategy is now being implemented in Montes Claros (MG, Brazil) (2 primary care centers have already been enrolled) and will be launched in Belo Horizonte and Nova Lima (MG - Brazil) in the following months, also including screening for other degenerative and congenital valve conditions. For this purpose, primary care physicians will also be trained in basic echocardiographic skills for routine evaluation of different age groups, utilizing the same telemedicine infrastructure for uploads and remote interpretation. We believe this integration is a crucial step for long-term sustainability of echocardiographic screening and for its integration into healthcare policies, and will allow assessment of the ideal screening strategies, including cost-effectiveness analysis. If RHD screening in these scenarios proves to be feasible and cost-effective, the final step would be to include it as a priority for the discussion of the Public Health System budget in the long run.
